# Editorial: Biologically active products as therapeutic options for the treatment of cardiovascular diseases related to liver injury

**DOI:** 10.3389/fphar.2022.1041020

**Published:** 2022-10-24

**Authors:** Arquimedes Gasparotto Junior, Francislaine Aparecida dos Reis Lívero, Alexandra Acco

**Affiliations:** ^1^ Federal University of Grande Dourados, Dourados, MS, Brazil; ^2^ Paranaense University, Umuarama, PR, Brazil; ^3^ Federal University of Paraná, Curitiba, PR, Brazil

**Keywords:** natural products, MAFLD (metabolic associated fatty liver disease), ethnomedicine, myocardia infarction, reverse cholesterol transport (RCT)

Cardiovascular and liver diseases are among the leading causes of morbidity and mortality worldwide. These conditions are complex problems, since heart diseases (chronic or acute heart failure) affect the liver and liver diseases affect the heart. With increasingly urbanized lifestyles and dietary changes involving high caloric contents, the overall prevalence of non-alcoholic fatty liver disease (NAFLD) has increased dramatically (Wei and Guo, 2022). Beyond NAFLD, liver disease afflicts over 10% of the world population (Mishra and Tiwari, 2011; Zhang et al., 2013), including chronic hepatitis, alcoholic steatosis, fibrosis, cirrhosis and hepatocellular carcinoma (HCC), which are the most health-threatening conditions drawing considerable attention from medical professionals and scientists. NAFLD is associated with metabolic syndrome and the development of cardiovascular diseases (Wei and Guo, 2022). Therefore, it is crucial to identify interactions between heart and liver, in order to provide the best treatment for both (Correale et al., 2018).

**FIGURE 1:**
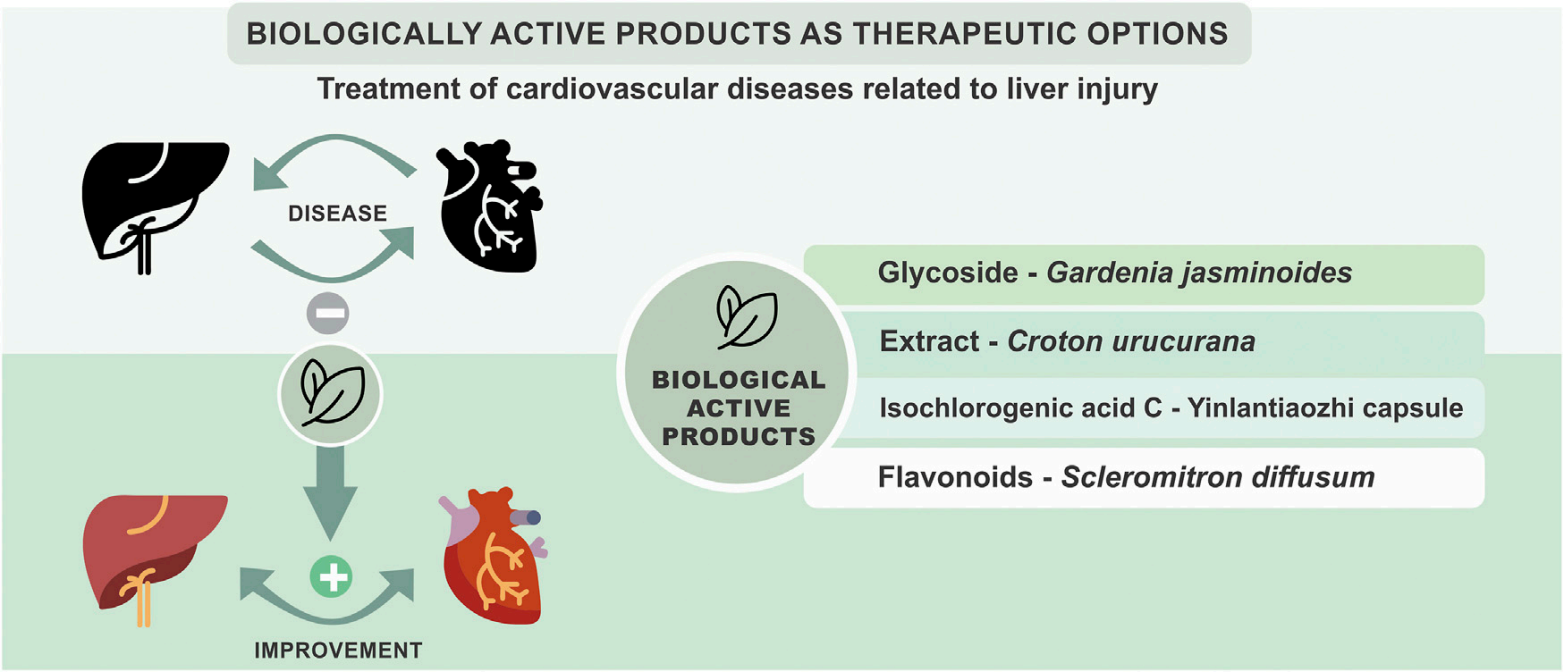
Relationship between liver and cardiovascular diseases and therapeutic possibilities presented in preclinical studies in this Research Topic, focusing on biologically active natural compounds.

Cardiac and hepatic diseases require effective and cost-efficient treatments (Wei and Guo, 2022). Biological active products can protect both liver and cardiovascular system. Herbs and herbal products have been used for the treatment of several ailments, since more than 2000 plants are known in ethnomedicine, and some of them are traditionally used to prevent or treat cardiovascular diseases and related complications (Patrignani et al., 2021). It is estimated that around 25% of currently commercialized medicines are derived from herbal plants used in traditional medicine (Sala et al., 2011; Patrignani et al., 2021), including those available for the therapy of liver or cardiovascular diseases, such as silymarin and naringenin, digoxin and captopril, respectively. In this special topic untitled *Biologically active products as therapeutic options for the treatment of cardiovascular diseases related to liver injury*, the authors presented original results of different natural products, obtained from several sources, on pre-clinical studies related to cardiovascular and hepatic diseases, as summarized in Figure 1. Shen et al. worked through the glycoside extracted from the dried mature fruit of *Gardenia jasminoides* J. Ellis; Auth et al. investigated the *Croton urucurana* extract, which main constituents are flavonoids, glycosides, and alkaloids; Zheng et al. investigated the isochlorogenic acid C from Yinlantiaozhi capsule; and Qin et al. worked with flavonoids extracted from *Scleromitron diffusum* (Willd.) R. J. Wang. Compared with the traditional methods of flavonoids extraction, Qin et al. established a bio-enzymatic method, which cell walls of the Chinese plant *S. diffusum* were more gently and thoroughly destroyed by cellulase to promote the out-flow of intracellular flavonoids, so that higher yields and more types of flavonoids could be harvested. These flavonoids showed significant hepatoprotective activity *in vitro* and *in vivo* in a rat model of NAFLD along 2 weeks of treatment. This article demonstrates the importance of explore the efficient extraction methods of flavonoids to improve the yields and to get their whole biological effects.

In a mice treatment lasted 12 weeks, complemented by *in vitro* cells study, Zheng et al. evaluated the effects of isochlorogenic acid C (ICAC) in a model of hyperlipidemia induced by high fat diet (HFD). Those authors focused in the increasing evidences that promoting reverse cholesterol transport (RCT) is an important strategy to treat hyperlipidemia. This elegant study revealed thought biochemical measurements, and gene and protein expression, that the ICAC acts in a role like synthetic LXR (liver X receptor) agonists, which promotes the whole-body RCT. However, unlike most LXR agonists, ICAC can treat hyperlipidemia without induce liver adipogenesis because it has no effect on liver LXRα. Furthermore, ICAC also impeded the inflammatory response in the liver, indicating that it could be a new RCT promoter for hyperlipidemia treatment without causing liver steatosis.

The study of Auth et al. investigated the effects of the *Croton urucurana* Baill extract in a complex *in vivo* model of metabolic associated fatty liver disease (MAFLD), a condition that in humans are related to several “hits” that accelerate the progression from hepatic steatosis to more advanced stages of the disease, including cirrhosis, liver failure, HCC, and cardiovascular disorders (Eslam et al., 2020). The MAFLD model included spontaneously hypertensive rats that received HFD (0.5% cholesterol-enriched diet) and were exposed to cigarette smoke (9 cigarettes/day for 10 weeks). The treatment with *C. urucurana* extract demonstrated a dose-dependent effect to control the MAFLD, including reduction of plasmatic lipids and transaminases, hepatic lipid droplets and oxidative stress biomarkers. These data suggest that this plant extract may be useful for treating patients with MAFLD, especially when associated with hypertension, smoking, and dyslipidemia.

Finally, the work of Shen et al. investigated the effects of geniposide (GEN) against the ferroptosis, a process present in cardiomyocytes cell death, caused by iron-dependent oxidative stress. This was an *in vitro* study that applied primary mice cardiomyocytes and rat cardiac H9c2 cells, complemented by an *in vivo* model of myocardial infarction (MI) in rats. The results indicate that ferroptosis induced by iron overload resulted in myocardial oxidative injury in cardiomyocytes, however the GEN treatment showed cardioprotection. It was found that activation of the Grsf1/GPx4 axis by GEN treatment inhibited myocardial ferroptosis and oxidative stress, suggesting that GEN could be a promising drug candidate for ischemic heart disease.

It should be stressed that all original researches reported in this Research Topic applied appropriated positive and negative controls, both *in vitro* and *in vivo*. This caution demonstrates the correct experiments designed by the authors and also increases the credibility of the scientific results herein presented.

Finally, this Edition provides a deep overview of the natural compounds as therapeutic tools for the treatment of cardiovascular diseases and hepatic injuries. Collectively, the pre-clinical studies that compose the Research Topic “Biologically Active Products as Therapeutic Options for the Treatment of Cardiovascular Diseases Related to Liver Injury” present perspectives for future researches regarding new therapies in cardiovascular and hepatic diseases.
